# Bibliography

**Published:** 1888-04

**Authors:** 


### BIBLIOGRAPHY.

DISEASES OF THE HEART AND LUNGS, by James R. Leaming, M. D., Emeritus Professor of Diseases of the Chest and Physical Diognosis in the New York Polyclinic ; and President of the Faculty, Special Consulting Physician in Chest Diseases, St. Luke's Hospital, New York, etc. Second edition revised and corrected.

The author of this treatise has made the diseases of the heart and lungs his special study for many years. His careful investigations as a Practitioner and Professor in New York, his observations in Public Hospitals and private consultations were occasionally embodied in papers, read before the Academy of Medicine or published in Medical Journals. These having been discussed, the views presented being sometimes, modified, strengthened or confirmed, were afterwards tested and in their revision, are given to the profession in this permanent form.

The book is therefore submitted to the profession as a valuable contribution to the fuller knowledge and treatment of diseased or abnormal conditions of the cardiac and respitory system. In one large octav volume, 300 pages. Price, $2.75, E. B. Treat, Publisher, 771 Broadway, New York.

---

A PRACTICAL TREATISE ON DISEASES OF THE SKIN. By John V. Shoemaker, A. M., M. D., Professor of Skin Diseases, in the Medico-Chirurgical College and Hospital of Philadelphia. Physician to the Philadelphia Hospital for diseases of the skin, etc., etc. D. Appleton & Co., N. Y., publishers, 1888, p. 632, royal octavo, cloth.

The name of the publishers is a sufficient guarantee for the mechanical appearance of the work.

The volume contains about 600 pages, well printed in large, clear type, and is illustrated with a number of fine colored plates.

The extensive experience the author has had in this particular branch, renders him competent to get out a book of the highest practical value to physicians and students.

A noticable and commendable feature of Prof. Shoemaker's work is the omitting of many cumbrous technicalities which only mislead and confuse the student, and substituting therefor words and phrases well understood and fully conveying the meaning intended.

The author modestly disclaims any special originality for the work further than the statement of the relative effects and values of numerous agents tested by him in his many years of clinical experince in the treatment of skin diseases. It is a valuable work, and its weak point, if it has one, is in its polytherapy.

---

THE PRESCRIPTION. Therapeutically, Pharmaceutically and Grammatically considered. By Professor O. A. Wall, M. D., Ph. G., Professor of materia medica and botany in the St. Louis College of Pharmacy.—Professor of Pharmacy in the Missouri Medical College, etc., etc., St. Louis, published by the Aug. Gast. Bank Note Co.—1888.

Correct prescription writing is a rare accomplishment. It is the best index to a medical man's attainments, and one can very generally estimate a physician's status, as to the extent of his cultivation by his prescriptions. This book will be found a valuable aid and guide to the acquirement of a correct style in writing prescriptions.

---

OBSTETRIC SYNOPSIS.—Physician's and Student's Ready Reference Series. By John S. Stewart, M. D., Demonstrator of Obstetrics and Chief Assistant in the Gynecological Clinic of the Medico-Chirurgical College of Philadelphia. Published by F. Davis, Phtladelphia, 1888.

This little volumn contains 188 pages and is well illustrated. It is especially designed to assist the undergraduate in acquiring a thorough knowledge of this department. It is systematic and to the point.

---

THE THREE ETHICAL CODES. Cloth, 55 pages, postpaid, 50 cents. The Illustrated Medical Journal Co., Publishers, Detroit, Mich.

In this little book is reprinted the Code of Ethics of the Amerioan Medical Association, with its Constitution, By-Laws and Ordinances, brought down to 1888; The Code of Ethics of the American Institute of Homœopathy and the Code of Ethics of the National Eclectic Medical Society. Of the three Codes, that of the American Medical Association is the longest, and that of the Eclectic Society is the shortest, while much of the Homœopathic is strikingly similar to that of the first mamed. Altogether, it is a handy litile book for reference as occasion may require.

---

A MANUAL OF MEDICAL JURISPRUDENCE, with reference to diseases and injuries of the nervous system, by Allan McLane Hamilton, M. D.; with illustrations. New York, 1887. E. B. Treat, 779 Broadway, publisher. Cloth, boards, price $2.75.

It is notorious that the subject of which this book treats is one which is too much neglected by the medical profession, considering the frequency with which physicians are placed upon the stand, and the great responsibility there resting upon them. It has been said in our hearing, by a learned judge, that "a shrewd lawyer can prove anything by the doctors." This is a slur, but it is not without some foundation; not that the witnesses are corrupt, but in the majority of cases, perhaps, they are ignorant of medical jurisprudence ; and especially where the nervous system is brought into prominence. There is no doubt that courts and physicians are often deceived by designing persons, feigning injury. It would be well if every physician, who expects at any time to be called to the witness stand, to testify, as an expert, could read this admirable work of Dr. Hamilton. His experience as a neurologist enables him to speak with authority, and his style is *lucid* and to the point. E. B. Treat, the publisher, is displaying commendable enterprise in bringing out works in the domain of medicine.

---

WEARING OF THE GRAY: Comprising Personal Portraits, Scenes and Adventures of the Late War.

With thrilling narratives of the daring deeds, dashing charges toilsome marches, willing sacrifices, and patient sufferings of the "Boys in Gray"; interspersed with stirring incidents of life in camp and hospital, and many important events hallowed by association with the gallant dead. By John Esten Cooke, formerly of General Stuart's staff, and author of "Surrey of Eagle's Nest," "Life of Stonewall Jackson," etc. Illustrated. E. B. Treat, 779 Broadway; Cincinnati Publishing Co., Cincinnati, O.; R. C. Treat, Chicago. Royal octavo; cloth and boards; 600 pages.

Wearing of the Gray has already taken its place in the affections of the Southern people; yet, to one who was present, and personally familiar with some of the scenes and incidents described in this thrilling work—familiar with localities and personally acquainted with the heroes—it has a charm indescribable. Poor Hardeman Stuart, he was our friend and companion and messmate, ere he was detailed on the signal corps. A tear to his memory. The style is fascinating, as in Surrey of Eagle's Nest; and one can scarcely put the book away till finished, so absorbing it is in every detail. Price, not given.

---

The name of that sterling journal, the "Mississippi Valley Medical Monthly," at Memphis, has been changed to the MEMPHIS MONTHLY MEDICAL JOURNAL.

---

THE BROOKLYN MEDICAL JOURNAL is the latest addition to the list, we believe, of monthly medical publications. It presents a handsome appearance, is well gotten up, and evinces vitality enough to stick. Success to it.

---

"THE ANNALS OF SURGERY," published by Chambers & Co., St. Louis, has been added to our exchange list. It is said to be a very "expensive" publication—to the publishers— but it is destined to do a world of good. We have been informed that it boasts a salaried editor in London and one in St. Louis. Table of contents for January and February very good—"fair to middling"—but nothing extraordinary ; good reading.

---

THE EFFICACY OF COCA ERYTHROXYLON.—Notes and comments by prominent physicians. Respectfully submitted to the medical profession. Cloth, gilt; Mariani, publisher, New York and Paris.

A neat little volume "submitted as an evidence of the esteem, in which 'Vin Mariani Erythroxylon Coca' is held," and as "an answer to the many venomous imputations of would-be competitors." Prof. Fauvel is quoted as saying "Coca is *the* tonic *par excellence*," and can be given for any length of time without producing constipation, an effect common to nearly all other tonics.

This book will be mailed to physicians free, on application, mentioning this JOURNAL. Address Mariani & Co., 127 5th Avenue, New York.

We are in receipt of No. 1, Vol. 1, of *The Climatologist*, a quarterly journal of sixty odd pages of first class reading matter. It is published at Washington, D. C., by William C. Chase, at the low price of 50 cents per year.

The Climatologist is devoted to climatotherapy, medical geography, epidemiology, demography, preventive medicine, and the investigation of mineral springs and health resorts.

Government, State, town and county health reports—domestic climate—agricultural climatology and chemistry—sanitary engineering—public and private water and gas works construction—irrigation—drainage—building, etc. It has seventy-four of the best known collaborators in this country. Judging from the number before us, it is a grand success to start with.

---

A neat pamphlet containing the proceedings of the State Medical Association of Florida, at St. Augustine, 1887, has been received. This was the thirteenth annual meeting of the Association. The pamphlet contains between 40 and 50 pages, on which were printed the roll of members, 72; minutes of two day's meeting; the prayer of the Chaplain; addresses of the Mayor and President, and two papers read before the Association, entitled "The Indian under Medical Observation," by Dr. Witt Webb, M. D., of St. Augustine, and "Was it Basilar Miningitis," by C. J. Kenworthy, M. D., M. R. S. V., of Jacksonville.

It is to be regretted that the profession in Florida is not better organized. The last proceedings, however, have the proper ring, and the meeting for 1888 is expected to be better attended.

